# Enhanced Corrosion Resistance of Carbon Steel in Hydrochloric Acid Solution by Eriobotrya Japonica Thunb. Leaf Extract: Electrochemical Study

**DOI:** 10.3390/ma10080956

**Published:** 2017-08-16

**Authors:** Wenjing Yang, Qihui Wang, Ke Xu, Yanjun Yin, Hebin Bao, Xueming Li, Lidan Niu, Shiqi Chen

**Affiliations:** 1 College of Chemistry and Chemical Engineering, Chongqing University, Chongqing 401331, China; qihuiwang@cqu.edu.cn (Q.W.); ke.xu@cqu.edu.cn (K.X.); yanjunyin@cqu.edu.cn (Y.Y.); reformasky@cqu.edu.cn (H.B.); 2 Chongqing Institute for food and Drug Control, Chongqing 401121, China; ldniu2@163.com (L.N.); chen1473@126.com (S.C.)

**Keywords:** carbon steel, Eriobotrya japonica Thunb., corrosion inhibitor

## Abstract

The biodegradable inhibitors, which could effectively reduce the rate of corrosion of carbon steel, were investigated by potentiodynamic polarization and electrochemical impedance spectroscopy (EIS). The mixed-type inhibitors extracted from Eriobotrya japonica Thunb. leaf exhibited excellent inhibition performance, and the inhibition efficiency for carbon steel reached 90.0% at 298 K in hydrochloric acid. Moreover, the adsorption mechanism of the inhibitors on a carbon steel surface is described by the Langmuir adsorption isotherm. Simultaneously, the corrosion morphology of the carbon steel and the inhibitor structure were analyzed by scanning electron microscope (SEM) and Fourier transform infrared spectroscopy (FT-IR), respectively.

## 1. Introduction

At present, various corrosion inhibitors such as inorganic and synthetic inhibitors have been employed in descaling and metal pickling processes [1,2]. However, their low bio-degradability and high cost severely limit their applications. Thus, the development of environmentally friendly, low-cost inhibitors is desirable and urgent [3,4]. Plant extracts are biodegradable in a natural environment, which allows them to serve as environmentally friendly inhibitors in acid solution [5]. Hydrochloric acid is one of the most widely used inorganic acids for metal pickling and descaling in industrial processes [6,7]. However, the acid is highly corrosive to metals and their alloys, especially for carbon steel (C-steel). Corrosion inhibitors have been extensively used to slow down the corrosion rate of C-steel [8]. Therefore, the development of plant extract inhibitors for C-steel pickling provides a new direction for these industrial processes. In recent years, green plant extracts of morus alba pendula [9], Justicia gendarussa [10], Zenthoxylum alatum [11], Salvia officinalis [12], Ginkgo [13], Musa paradisica [14], Tagetes erecta [15], Gum arabic [16], Artemisia pallens [17], lupine [18], Jasminum nudiflorum Lindl. [19], Dendrocalamus brandisii [20], aqueous garlic peel [21], henna [22], Nypa fruticans Wurmb [23], Damsissa [24], Mentha pulegium [25], olive [26], and Nigella sativa L. [27] have been widely studied and examined as effective inhibitors. However, to our knowledge, the corrosion inhibition behavior of Eriobotrya japonica Thunb. leaf extract (EJTLE) on C-steel in hydrochloric solution has not yet been reported.

Eriobotrya japonica Thunb. belongs to the genus Rosa, which is prevalent in most provinces of southern China. The fruit of this plant, one of the important subtropical fruits, and its leaves have been used medicinally to treat coughs, colds, chronic bronchitis, phlegm, high fever, and gastro-enteric disorders [28].

**Figure 1:**
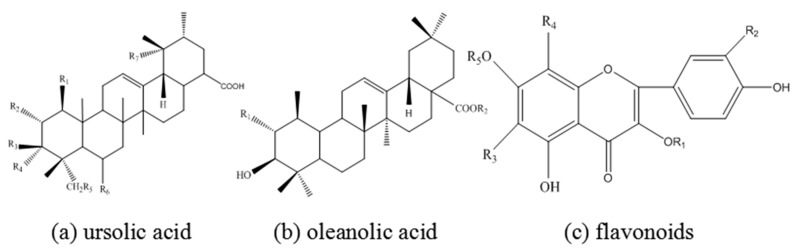
Molecular structures of (**a**) ursolic acid; (**b**) oleanolic acid; and (**c**) flavonoids.

The chemical composition of EJTLE largely consists of ursolic acid, oleanolic acid, and flavonoids. The molecular structures of ursolic acid [29], oleanolic acid, and flavonoids [30] are shown in Figure 1. These chemical constituents have anti-corrosion effects due to their heterocyclic structures and have been extensively researched. In general, these organic compounds contain heteroatoms such N, S, and O and are classified as heterocyclic compounds, which are regarded as effective adsorption centers.

In this work, the corrosion effect of EJTLE on C-steel was investigated by potentiodynamic polarization, electrochemical impedance spectroscopy (EIS), Fourier transform infrared spectroscopy (FT-IR), and scanning electronic microscopy (SEM). The adsorption behavior of EJTLE on the surface of carbon was also investigated.

## 2. Results

### 2.1. Open Circuit Potential Measurements

**Figure 2:**
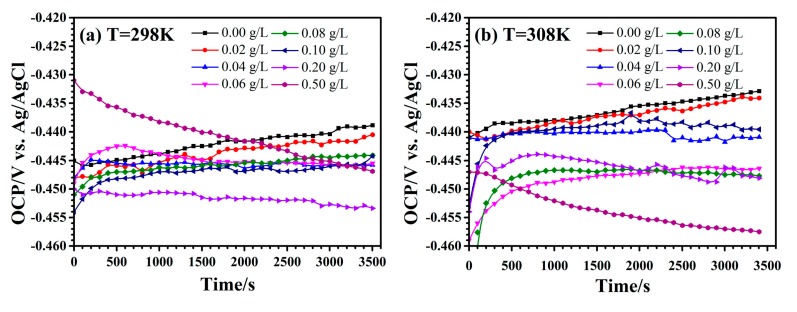

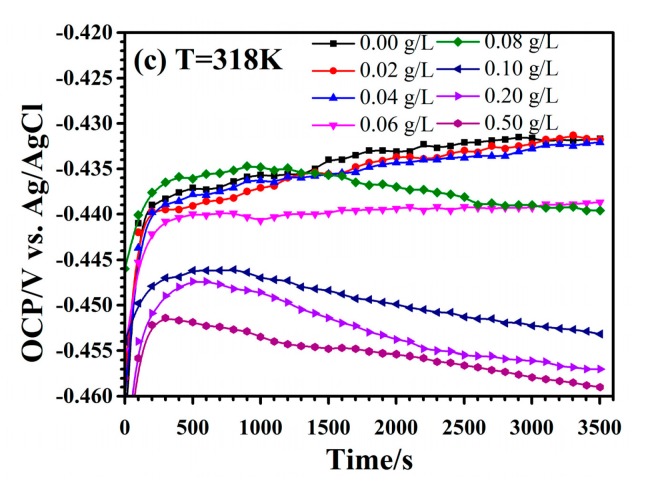
Open circuit potential curves of C-steel in 1 M HCl solution with different concentrations of Eriobotrya japonica Thunb. leaf extract (EJTLE) at (**a**) 298 K; (**b**) 308 K; and (**c**) 318 K.

Figure 2 shows the open circuit potential curves of C-steel in 1 M HCl solution with different concentrations of EJTLE at different temperatures prior to electrochemical analysis. In Figure 2, the shape of the inhibition curves is similar to that of the uninhibited ones, suggesting that lower concentrations (below 0.06 g·L^−1^) of inhibitors did not significantly change the corrosion behavior of C-steel. Interestingly, the open circuit potential curve of carbon steel (C-steel) increases at first and then slowly decreases in the acid solution with higher concentrations of corrosion inhibitors (above 0.06 g·L^−1^). This phenomenon is related to the adsorption of inhibitors on the C-steel surface. The inhibitors contain heteroatoms and π bonds adsorbed on the electrode surface, resulting in higher electron density on the C-steel surface and negative electrode potential.

### 2.2. Polarization Measurements

**Figure 3:**
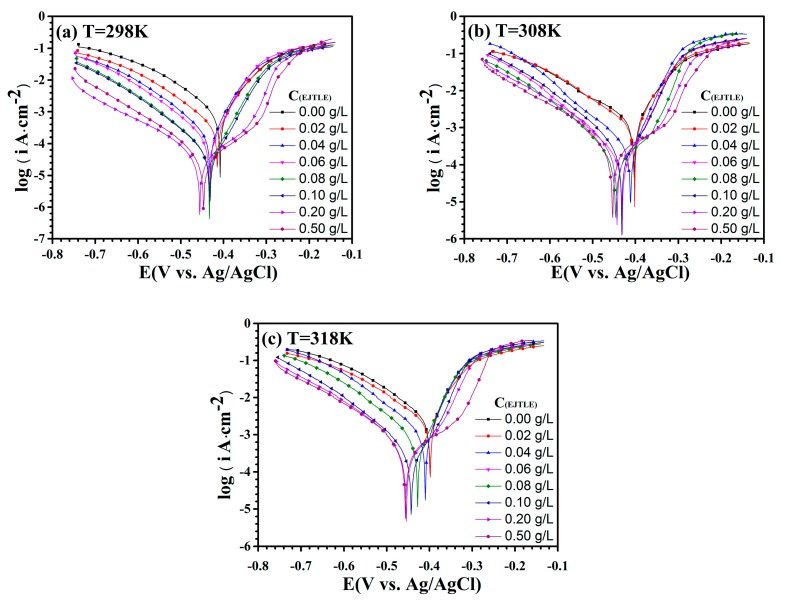
Polarization curves of C-steel in 1 M HCl solution with different concentrations of EJTLE at (**a**) 298 K; (**b**) 308 K; and (**c**) 318 K.

The polarization curves of C-steel in 1 M HCl solution at different temperatures are shown in Figure 3. As shown in Figure 3, the corrosion potential of C-steel in 1 M HCl acid is obviously negative, with an increasing concentration of corrosion inhibitors. In addition, the cathodic and anodic polarization curves exhibit low current density, meaning that the cathodic and anodic polarization reactions were inhibited. In order to show clearly the mechanism of corrosion inhibitors, cathodic polarization and anodic polarization are discussed separately. Compared with those of the blank solution, the cathodic polarization curves of C-steel with corrosion inhibitors did not change significantly. Similarly, the anodic polarization curves of C-steel with inhibitors were lower than the anodic polarization curves of C-steel in the blank solution. Additionally, the current plateaus in the anodic polarization curve, as expected.

### 2.3. Electrochemical Impedance Spectroscopy

**Figure 4:**
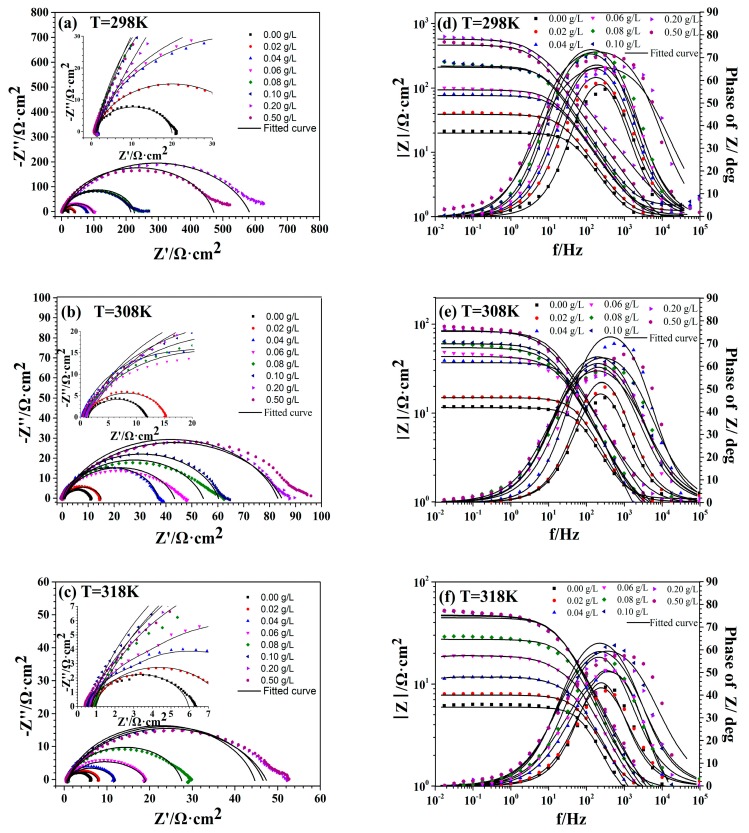
Nyquist plots (**a**) and Bode plots (**b**) of C-steel in 1 M HCl solution with different concentrations of EJTLE at 298 K; Nyquist plots (**c**) and Bode plots (**d**) of C-steel in 1 M HCl solution with different concentrations of EJTLE at 308 K; Nyquist plots (**e**) and Bode plots (**f**) of C-steel in 1 M HCl solution with different concentrations of EJTLE at 318 K.

The Nyquist impedance plots and Bode plots of C-steel in 1 M HCl solution with and without different concentrations of EJTLE are shown in Figure 4. After the addition of inhibitors, the Nyquist and Bode diagrams of C-steel were similar to the Nyquist and Bode diagrams of the blank corrosion solution and only occured as capacitive arcs. The capacitive arc reflects the relaxation process of the interface double layer and the charge transfer resistance. The diameter of the capacitive arc increases with increasing inhibitor concentration, which indicates that the corrosion of the C-steel electrode in 1 M HCl solution is inhibited. In combination with the corresponding Bode diagram, this means that the inhibited corrosion of C-steel was controlled by a time constant. Obviously, there is no Warburg impedance or inductive reactance arc in Nyquist plots.

**Figure 5:**
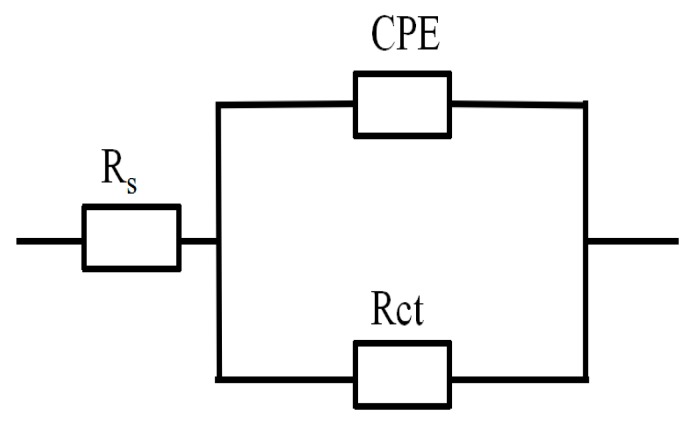
The equivalent circuit model used to fit the electrochemical impedance spectroscopy (EIS) data.

**Figure 6:**
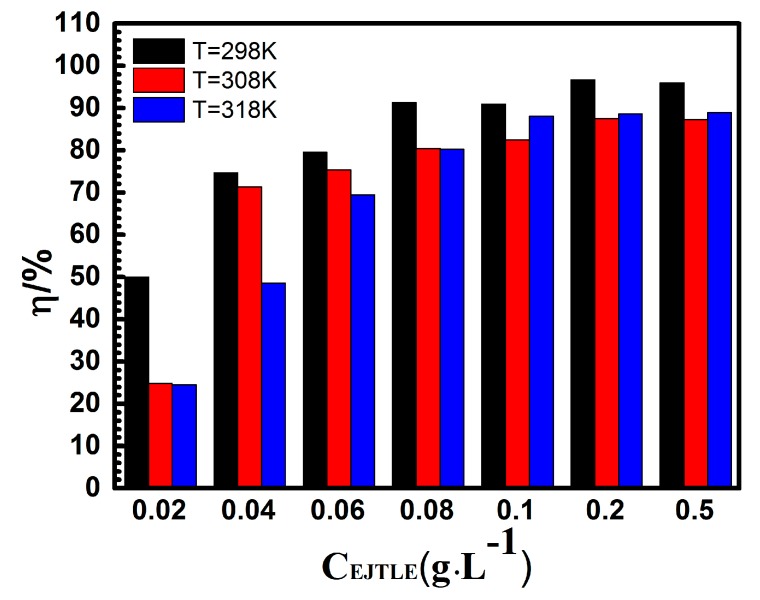
Variation of η in the presence of different concentrations of EJTLE at 298 K, 308 K, and 318 K.

We used the equivalent circuit model shown in Figure 5 to fit the data results of the electrochemical impedance spectroscopy (EIS). Figure 6 shows the variation of inhibition efficiency (η) in the presence of different concentrations of EJTLE at 298 K, 308 K, and 318 K. The inhibition efficiency of the plant inhibitors increased with increasing concentration. Additionally, the inhibition efficiency of the plant extract decreased as temperature increased. The results show that the plant inhibitors exhibit enhanced anti-corrosion performance, which can be used for high-efficient inhibitors in C-steel pickling and descaling processes.

### 2.4. Adsorption Isotherm

**Figure 7:**
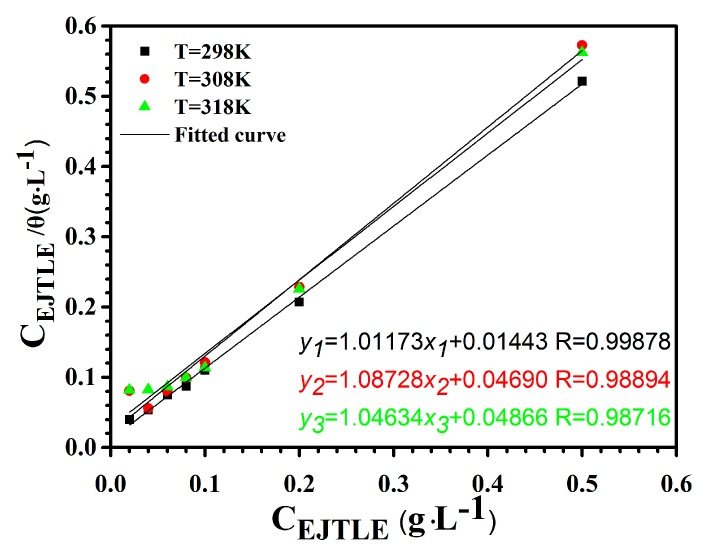
Langmuir adsorption isotherm of EJTLE on the C-steel surface in 1 M HCl solution at 298 K, 308 K and 318 K.

Generally, organic inhibitors have been accomplished by the adsorption of a protective film at the metal-solution interface, and the mechanism can be described by an adsorption isotherm. Figure 7 represents the fitting of C/θ versus C with a linearly dependent coefficient near 1.

### 2.5. FT-IR Results

**Figure 8:**
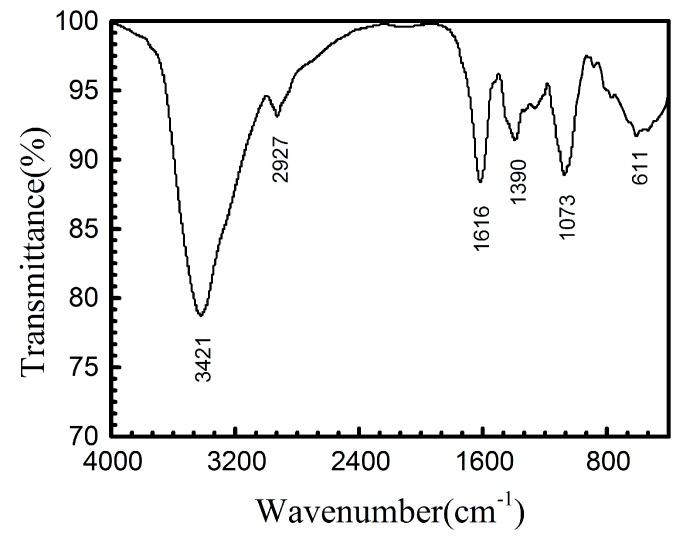
Fourier transform infrared spectroscopy (FT-IR) spectra of Eriobotrya japonica Thunb. leaf extract (EJTLE).

In order to characterize and confirm the product structure, FT-IR spectroscopy was performed. The FT-IR spectrum of EJTLE powder is shown in Figure 8. The strong band at 3421 cm^−1^ corresponds to the N–H or O–H stretching vibration. The band at 2927 cm^−1^ is related to the –CH_2_ asymmetrical stretching vibration. The band at 1616 cm^−1^ is attributed to the C=C stretching vibration. The band at 1390 cm^−1^ could be assigned to the C–H bending in –CH_3_. The adsorption band at 1073 cm^−1^ corresponds to the C–N or C–O stretching vibration. The band at 611 cm^−1^ can be assigned to C–H of aliphatic and aromatic carbon.

### 2.6. SEM Analysis

**Figure 9:**
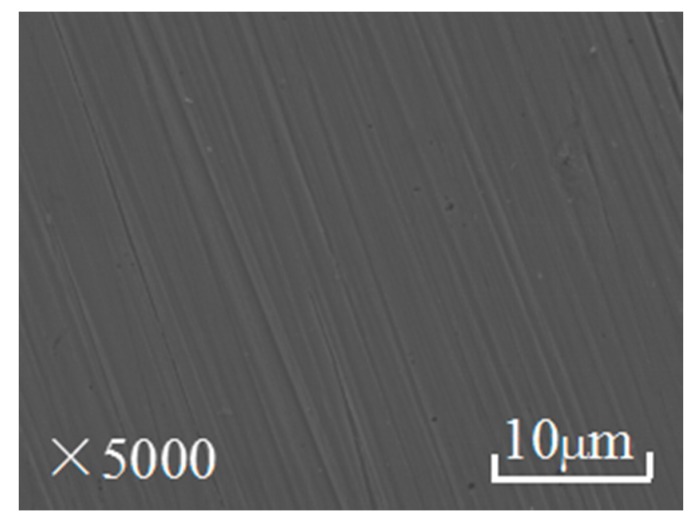
SEM images of the C-steel surface after sanding.

**Figure 10:**
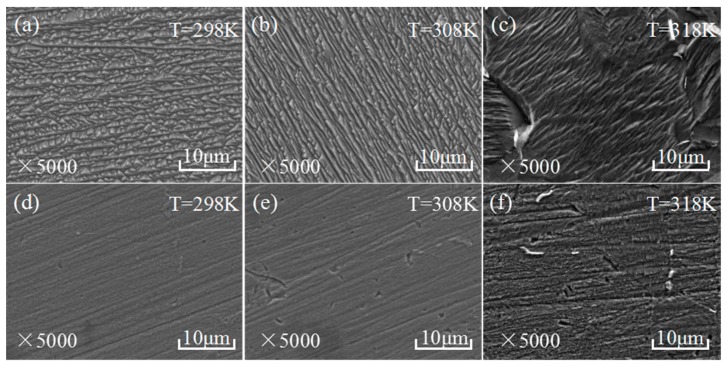
SEM images of the C-steel surface after immersion in 1 M HCl for 1 h: (**a**) at 298 K; (**b**) at 308 K; (**c**) at 318 K; (**d**) containing 0.5 g·L^−1^ EJTLE at 298 K; (**e**) containing 0.5 g·L^−1^ EJTLE at 308 K; and (**f**) containing 0.5 g·L^−1^ EJTLE at 318 K.

It can be seen from Figure 9 that the polished C-steel surface is smooth and without obvious scratches. Figure 10 shows the surface graphs of the C-steel surface exposed to 1 M HCl in the absence and presence of 0.5 g·L^−1^ EJTLE at three temperatures. It can be clearly seen from Figure 10a–c that the rough surface with several grooves was caused by uniform corrosion in acid solution. Conversely, in the presence of 0.5 g·L^−1^ plant inhibitors, the surface (Figure 10d–f) of C-steel shows relatively smooth polishing scratches. In light of these findings, we can conclude that the plant inhibitors do prevent the corrosion of carbon steel in 1 M HCl solution by the formation of adsorbed molecules on the surfaces of C-steel specimens.

## 3. Discussion

An extract was obtained from Eriobotrya japonica Thunb. leaves by immersing them in hot water at 363 K for 1 h. The properties of the extract as a corrosion inhibitor in an acid pickling solution were investigated by electrochemical experiments, SEM, and FT-IR.

In Figure 3, polarization curves show that the inhibitors can be considered mixed-type corrosion inhibitors because the corrosion potential was less than 85 mV [31,32]. Moreover, the plateau of the anodic polarization curves shows that the inhibitors desorbed from the C-steel surface. It is generally believed that this phenomenon indicates the formation of an adsorbed layer on the surface of C-steel, inhibiting cathodic hydrogen evolution and anodic iron dissolution. The inhibition efficiency can be calculated from the following equation:

(1) $$\eta \left(\%\right)=\left(\frac{I_{\mathrm{corr}}^{0}-I_{\mathrm{corr}}}{I_{\mathrm{corr}}^{0}}\right)\times 100$$

where $I_{\mathrm{corr}}$ and $I_{\mathrm{corr}}^{0}$ are the corrosion current densities of C-steel with and without plant inhibitors, respectively.

The corrosion parameters calculated from Figure 3 are shown in Table 1. With increasing concentrations of plant extract, the corrosion potential of the electrode gradually shifted to a negative range of about 50 mV. When the inhibitor concentration was 0.1 g·L^−1^, the corrosion current density was 43.11 μA·cm^−2^ at 298 K, and the corrosion inhibition efficiency reached 95.7%. When the temperature increased to 318 K, the corrosion inhibition efficiency was still above 85.0%. The inhibition efficiency of this plant inhibitor is relatively better than that reported in the literature. Obviously, the results show that the proper concentration of inhibitors (over 0.1 g·L^−1^) can significantly reduce the corrosion reaction during the pickling process of C-steel. Moreover, the protective mechanism of the extract is to form an absorbed layer on the C-steel surface to inhibit the cathodic reduction reaction of H^+^ and the anodic dissolution reaction of iron.

Similarly, the EIS experiments also confirmed the results of the polarization experiments. The equivalent circuit model shown in Figure 5 was used to fit the EIS test parameters. As shown in Figure 5, *R*_s_ is the solution resistance, *R*_ct_ is the resistance of charge transfer, and the constant phase elements (CPE) are used to replace capacitance. The impedance values of CPE for the C-steel electrode in 1 M HCl solution is expressed as follows [8]:

(2) $$Z_{\mathrm{CPE}}=\frac{1}{Y_{0}{\left(j\omega \right)}^{n}}$$

In this expression, *Y*_0_ is a proportional factor, *ω* represents the angular frequency (*ω* = 2*πf*), n is the deviation parameter, and j is the imaginary unit. The capacitance of the electric double layer can be described as follows:

(3) $$C_{\mathrm{dl}}=\frac{Y_{0}\omega^{n-1}}{\sin\left(n\pi /2\right)}$$

The values of the inhibition efficiency can be calculated from *R*_ct_ by the following relationship:

(4) $$\eta \left(\%\right)=\left(\frac{R_{\mathrm{ct}}-R_{\mathrm{ct}}^{0}}{R_{\mathrm{ct}}}\right)\times 100$$

where $R_{\mathrm{ct}}$ and $R_{\mathrm{ct}}^{0}$ are the values of charge transfer resistance in 1 M HCl solution with and without EJTLE inhibitors, respectively.

The impedance parameters were obtained from the EIS measurements and the inhibition efficiency of C-steel in 1 M HCl solution with and without different concentrations of EJTLE, which are listed in Table 2. Specifically, when the inhibitor concentration is 0.1 g·L^−1,^ the charge transfer resistance increases from 19.02 Ω to 129.65 Ω in the blank solution at 298 K. Additionally, the double-layer capacitance is reduced from 198.3 F·cm^−2^ to 92.8 F·cm^−2^. This phenomenon indicates the formation of an adsorbed layer, which slows the rate of carbon corrosion. The results show that the plant inhibitors have enhanced corrosion resistance and can be used as efficient inhibitors for the pickling and descaling of C-steel.

Base on the *θ* values obtained from the EIS measurements, the mechanism of the EJTLE inhibitors on the C-steel in 1 M solution can be described by the Langmuir adsorption model [33]:

(5) $$\frac{C_{\mathrm{inh}}}{\theta }=\frac{1}{K_{\mathrm{ads}}}+C_{\mathrm{inh}}$$

where *K*_ads_ is the adsorption equilibrium constant, *C*_inh_ is the inhibitor concentration, and *θ* (*η*/100) is the coverage of the adsorbed inhibitors on the C-steel. The linearized mathematical expression has been obtained by the experimental data as follows:

(6) $$y_{1}=1.01173x_{1}+0.01443\;R^{2}=0.99878$$

(7) $$y_{2}=1.08728x_{2}+0.04690\;R^{2}=0.98894$$

(8) $$y_{3}=1.04634x_{3}+0.04866\;R^{2}=0.98716$$

Here, *y* and *x* represent the values of *C*_inh_/*θ* and *C*_inh_, respectively. The adsorption equilibrium constants obtained from the above-mentioned fitting relationship are 70.11 L·g^−1^, 23.18 L·g^−1^, and 21.50 L·g^−1^, respectively. Moreover, the standard adsorption free energy ($\Delta G_{\mathrm{ads}}^{0}$) is calculated by the following formula [34,35,36]:

(9) $$K_{\mathrm{ads}}=\frac{1}{55.5}\exp\left(\frac{-\Delta G_{\mathrm{ads}}^{0}}{RT}\right)$$

where *K*_ads_ is also the adsorption equilibrium constant, R is the gas constant, and *T* is the absolute temperature, respectively. The values of the standard adsorption free energy obtained from the calculation was −20.48 kJ·mol^−1^, −18.33 kJ·mol^−1^, and −18.73 kJ·mol^−1^, respectively. Namely, the results indicated that the type of adsorption of the plant inhibitors on the C-steel surface is physical absorption.

The FT-IR results explain the physical adsorption of the inhibitors on the surface of C-steel. Figure 8 shows that the Eriobotrya japonica Thunb. leaf extracts contain C, N, and O atoms in functional groups O–H, N–H, C=C, C–H, C–N, and C–O. These functional groups are effective adsorption centers that adsorbed on the C-steel surface and increased the charge transfer resistance to further retard the corrosion of C-steel.

According to the relevant literature, Eriobotrya japonica Thunb. leaves contain ursolic acid, oleanolic acid, and flavonoids. The structures of these molecules contain heteroatoms and benzene rings. The incompletely filled d electron orbitals of iron can interact with the lone electrons of polar groups in the inhibitors. The corrosion inhibitors form a compact protective film on the surface of C-steel, thus slowing down the corrosion rate of C-steel in HCl solution.

The use of extracts of highly prevalent plants may be an environmentally friendly and economical way to produce corrosion inhibitors. This method not only avoids the use of non-biodegradable, environmentally harmful inhibitors but it can also provide an efficient use for plants. As a result, researchers have studied plant extract inhibitors, and the results show that some natural plant extracts have significant corrosion inhibition efficiency. However, few studies have investigated the chemical constituents of plant extracts. There are many kinds of chemical constituents in plant extracts, and their compositions are quite different. It is worth mentioning that the properties of some components in the inhibitors may change in the acid solution. It is difficult to accurately analyze the composition of plant extracts and inhibitors. Therefore, even though experiments have proved that some plant extracts have high corrosion inhibition efficiency, there will still be doubts about the rationality of plant extract inhibitors.

Therefore, researchers should pay more attention to the inhibition effect of the main components in the extracts and explore the influence of the inhibitor extraction and preservation methods on the inhibition efficiency. Consequently, a deeper theoretical analysis should be carried out for corrosion inhibitors. In this paper, we conclude that Eriobotrya japonica Thunb. leaf extracts have a good corrosion inhibition effect on C-steel. The results of this study will provide sound scientific references for future studies.

## 4. Materials and Methods

### 4.1. Inhibitors and Electrode Preparation

Fresh Eriobotrya japonica Thunb. leaves were rinsed with tap water, dried in an oven at 323 K for 24 h, and ground into powder. A sample of 37.45 g of powdered leaves was dispersed in 1.0 L water and heated at 363 K for 1 h. The mixture was filtered, and a clear brown solution was dried in an oven at 353 K to obtain 5.31 g of powder.

Q235 C-steel (0.14% C, 0.19% Si, 0.55% Mn, 0.028% P, 0.020% S, and Fe balance) specimens, with dimensions of 1.0 cm × 1.0 cm × 0.3 cm and 0.5 cm × 0.5 cm × 0.3 cm were prepared for electrochemical and surface characterization studies. All metal specimens were polished carefully with emery paper (grade 400-600-800-1000, MATADOR, Remschei, Germany) and rinsed with distilled water and ethanol before the experiment. The HCl solution was prepared by diluting a concentrated HCl solution (37%) with distilled water. The concentration ranges of EJTLE varied from 0.02 to 0.5 g·L^−1^.

### 4.2. Electrochemical Measurements

All electrochemical experiments were performed with a CHI660B electrochemical workstation (CHI Co., Shanghai, China) in a conventional three-electrode cell system with Q235 C-steel (1.0 cm × 1.0 cm) embedded in epoxy resin holders as the working electrode (WE), a platinum gauze as the counter electrode (CE), and a silver-silver chloride (Ag/AgCl) electrode (saturation concentration KCl) with a Luggin-Haber capillary as the reference electrode (RE). In this test, all electrochemical experiments were conducted after the metal specimens were immersed in 1 M HCl solution at open circuit potential (OCP) for 1 h until a steady state was obtained. The potentiodynamic polarization experiments employed the following parameter: scanning from −300 to +300 mV (versus OCP) at a scanning rate of 0.5 mV·s^−1^. The electrochemical impedance spectra were measured at OCP with 10 mV AC amplitude over a frequency range of 100 kHz to 10 mHz. EIS data analysis was performed via ZView 3.0 version software (Scribner Associates Inc., Southern Pines, NC, USA).

### 4.3. Morphology Analysis

The corrosion microscopy of the C-steel surface before and after immersion in 1 M HCl solution with and without 0.5 g·L^−1^ EJTLE at different temperatures for 1 h was characterized by TESCAN MIRA 3 (TESCAN, Brno, Czech Republic) scanning electronic microscopy (SEM). The accelerating voltage for SEM was 15 kV. In addition, FT-IR analysis (KBr pellet method) was performed with a Nicolet iS50 FT-IR Spectrometer (Thermo Fisher Scientific, Waltham, MA, USA) to characterize the product structure of EJTLE.

## 5. Conclusions

In this paper, electrochemical and surface characterization experiments were conducted to investigate the anti-corrosion effects of Eriobotrya japonica Thunb. leaf extracts as corrosion inhibitors for carbon steel in a hydrochloric acid pickling solution. The results show that the inhibitors form a barrier layer on the carbon steel surface by physical adsorption, thus inhibiting the cathodic hydrogen evolution and anodic dissolution reactions. Moreover, Eriobotrya japonica Thunb. leaf extracts are effective mixed-type corrosion inhibitors.

## Figures and Tables

**Table 1:** Polarization parameters for C-steel in 1 M HCl solution with different concentrations of EJTLE at different temperatures.

Temperature (K)	CEJTLE (g·L^−1^)	Ecorr (mV vs. Ag/AgCl)	Bc (mV·dec^−1^)	Ba (mV·dec^−1^)	*I*_corr_ (uA·cm^−2^)	*η* (%)
298	0	−408	−109	80	1006.00	-
	0.02	−415	−146	77	597.90	40.6
	0.04	−431	−96	67	229.50	77.2
	0.06	−433	−116	54	145.60	85.5
	0.08	−433	−92	50	47.92	95.2
	0.1	−432	−95	51	43.11	95.7
	0.2	−456	−76	91	28.88	97.1
	0.5	−447	−74	100	30.64	97.0
308	0	−403	−176	71	1864.00	-
	0.02	−402	−131	53	1194.00	35.9
	0.04	−411	−117	44	287.30	84.6
	0.06	−426	−106	53	229.20	87.7
	0.08	−433	−105	59	189.20	89.9
	0.1	−432	−107	59	206.20	88.9
	0.2	−443	−115	121	250.20	86.6
	0.5	−453	−92	157	245.90	86.8
318	0	−398	−121	52	2826.00	-
	0.02	−398	−120	47	2326.00	17.7
	0.04	−410	−117	48	1007.00	64.4
	0.06	−428	−120	45	546.00	80.7
	0.08	−429	−114	49	337.60	88.1
	0.1	−443	−108	58	265.50	90.6
	0.2	−454	−114	92	360.90	87.3
	0.5	−456	−123	125	461.60	83.7

**Table 2:** The impedance parameters for C-steel in 1 M HCl solution with and without various concentrations of EJTLE at different temperatures.

Temperature (K)	CEJTLE (g·L^−1^)	*R*_s_ (Ω·cm^−2^)	*R*_ct_ (Ω·cm^−2^)	CPE-T/*Y*_0_ × 106 (Ω^−1^·cm^−2^ s^n^)	*n*	*C*_dl_ (uF·cm^−2^)	*η* (%)	*f*
298	0	0.98	19.02	290.95	0.93	198.3	-	37.56
	0.02	1.03	38.01	320.12	0.89	188.1	50.0	21.23
	0.04	1.23	75.25	217.26	0.89	128.1	74.7	21.23
	0.06	0.96	92.81	224.94	0.87	132.8	79.5	11.91
	0.08	1.07	219.20	143.46	0.91	102.4	91.3	8.071
	0.1	1.49	208.50	129.65	0.91	92.8	90.9	6.643
	0.2	1.02	573.00	123.90	0.78	66.6	96.7	3.742
	0.5	0.69	463.60	122.92	0.86	80.7	95.9	3.742
308	0	1.09	10.48	440.74	0.90	238.8	-	81.38
	0.02	1.01	13.94	347.00	0.91	196.4	24.8	81.38
	0.04	0.30	36.62	208.90	0.93	145.2	71.4	25.7
	0.06	0.77	42.60	491.82	0.82	229.7	75.4	14.36
	0.08	0.66	53.85	520.29	0.81	233.3	80.5	14.36
	0.1	0.62	59.74	434.35	0.84	236.7	82.5	8.071
	0.2	0.95	83.82	499.15	0.77	214.3	87.5	8.071
	0.5	0.59	82.66	351.67	0.81	174.3	87.3	8.071
318	0	0.73	5.21	576.40	0.93	372.8	-	81.38
	0.02	0.89	6.91	675.58	0.88	316.1	24.5	81.38
	0.04	0.63	10.13	564.93	0.89	310.3	48.6	46.5
	0.06	0.77	17.00	595.16	0.84	251.0	69.4	46.5
	0.08	0.95	26.48	416.01	0.86	206.1	80.3	25.7
	0.1	0.66	43.92	383.54	0.85	200.5	88.1	14.36
	0.2	0.74	45.80	463.03	0.81	206.0	88.6	14.36
	0.5	0.44	46.90	547.17	0.77	211.0	88.9	14.36
